# Declining Trajectories of Co-occurring Psychopathology Symptoms in Attention-Deficit/Hyperactivity Disorder and Autism Spectrum Disorder: A 10-Year Longitudinal Study

**DOI:** 10.3389/fpsyt.2021.724759

**Published:** 2021-10-14

**Authors:** Stian Orm, Merete Glenne Øie, Ingrid Nesdal Fossum, Per Normann Andersen, Erik Winther Skogli

**Affiliations:** ^1^Division Mental Health Care, Innlandet Hospital Trust, Brumunddal, Norway; ^2^Department of Psychology, University of Oslo, Oslo, Norway; ^3^Research Department, Innlandet Hospital Trust, Brumunddal, Norway; ^4^Department of Psychology, Inland Norway University of Applied Sciences, Lillehammer, Norway

**Keywords:** attention-deficit/hyperactivity disorder (ADHD), autism spectrum disorder (ASD), psychopathology, longitudinal analyses, externalizing and internalizing behavior, neurodevelopmental disorders, adult outcomes

## Abstract

**Objective:** Our objective was to examine developmental trajectories of co-occurring psychopathology symptoms from childhood to young adulthood in individuals with Attention-Deficit/Hyperactivity Disorder (ADHD), individuals with Autism Spectrum Disorder (ASD), and typically developing (TD) individuals.

**Method:** We assessed co-occurring psychopathology symptoms in 61 individuals with ADHD, 26 with ASD, and 40 TD individuals at baseline (T1; M_age_ = 11.72, 64% boys), 2-year follow up (T2; M_age_ = 13.77), and 10-year follow up (T3; M_age_ = 21.35). We analyzed trajectories of internalizing behaviors, externalizing behaviors, and total problems with linear mixed models.

**Results:** From T1 to T3, the ADHD group displayed a small decline in internalizing behaviors (*d* = −0.49) and large declines in externalizing behaviors (*d* = −0.78) and total problems (*d* = −0.71). The ASD group displayed large declines in internalizing behaviors (*d* = −0.79), externalizing behaviors (*d* = −0.80), and total problems (*d* = −0.89). From T1 to T2, the decline in externalizing behaviors and total problems were significantly smaller in the ADHD group compared with the ASD group. The ADHD and the ASD group displayed more co-occurring symptoms compared with the TD group at T3.

**Conclusion:** Individuals with ADHD and ASD, respectively, displayed declines in co-occurring symptoms from childhood to young adulthood. Individuals with ASD displayed an earlier decline compared with individuals with ADHD. Compared with TD individuals, individuals with ADHD and ASD, respectively, continued to display elevated levels of co-occurring symptoms in young adulthood.

## Introduction

Attention-Deficit/Hyperactivity Disorder (ADHD) and Autism Spectrum Disorder (ASD) are two major neurodevelopmental disorders (1–3). Despite ADHD and ASD being separate diagnoses, they often co-occur (4–6) and share genetic risk and endophenotypes (7–10). Individuals with ADHD and individuals with ASD experiences high levels of comorbid disorders and co-occurring psychopathology symptoms across the lifespan (11–17).

The co-occurring symptoms may be of particular importance, as these symptoms predict poorer adult outcome, lower occupational functioning, and impaired quality of life in individuals with ADHD and individuals with ASD (18–24). Two broad dimensions of co-occurring symptoms are internalizing (e.g., anxiety and depression symptoms) and externalizing behaviors (e.g., rule-breaking behavior, conduct problems). Several factors contribute to high levels of co-occurring symptoms among individuals with ADHD and individuals with ASD, among them are executive dysfunction, symptom severity, and emotional dysregulation (25–28). However, little is known about the developmental trajectories of co-occurring symptoms in these two clinical populations and how the trajectories compare with each other and with typically developing (TD) individuals (29, 30).

### Development of Co-occurring Symptoms in ADHD

High levels of co-occurring symptoms are found across the lifespan in individuals with ADHD (16, 31, 32). In the longitudinal Massachusetts General Hospital Study of individuals with ADHD, the authors found that most comorbid disorders had an early onset in preadolescence years (33–36). In their 16-year follow-up, the authors found high life-time prevalence of comorbid disorders, but in young adulthood the ADHD group only presented with more anxiety disorders compared with the TD group (34). This could suggest that comorbid disorders, and hence co-occurring symptoms, wane over time in individuals with ADHD. In the longitudinal Berkley Girls Study, co-occurring symptoms were examined in childhood, adolescence, and young adulthood (37). The authors found children with ADHD to display more co-occurring symptoms in childhood (mean age 9; *d* = 0.94–1.69) (38), adolescence (mean age 14; *d* = 0.87–1.29) (39), and young adulthood (mean age 19; *d* = 0.77–1.30) (32) compared with TD individuals. These results suggest that individuals with ADHD consistently presents with more co-occurring symptoms from childhood to young adulthood. At the same time, effect sizes could suggest a slightly decline from childhood to young adulthood but no conclusion can be drawn due to the lack of longitudinal analyses of change over time.

A few studies have examined the developmental trajectories of co-occurring symptoms in individuals with ADHD. In a previous study by our research group, declines in internalizing and externalizing behaviors over a 2-year period (mean age 11–13) were found (40). However, in contrast, another longitudinal study following children with ADHD over a 3-year period (mean age 10–13) reported that the majority displayed persistent trajectories of internalizing and externalizing behaviors (61.5 and 93%, respectively) over a 3-year period, with only 10 and 7%, respectively, displaying declining trajectories (41). In a third study, following up children with ADHD 13-years later (mean age 25), the authors found declines in internalizing and externalizing behaviors compared with baseline scores (42). Thus, two studies have suggested declining trajectories of internalizing and externalizing behaviors (40, 42), whereas one study have suggested stable trajectories for the majority of individuals with ADHD (41). Despite a possible decline in co-occurring symptoms over time, the results of Hinshaw et al. (32) and Skogli et al. (40) suggests that individuals with ADHD continue to display high levels of co-occurring symptoms compared with TD individuals.

Some limitations of the aforementioned studies should be mentioned. First, two studies did not examine developmental trajectories but only differences between individuals with ADHD and TD individuals within different assessment waves (35–37). Second, two of three studies examining developmental trajectories only included two assessment waves (40, 42), preventing them from detecting change in the trajectories. Third, two of the three studies examined developmental trajectories over a short time-span (2 or 3 years) (40, 41). Fourth, the only study examining developmental trajectories over a long time-span, from childhood to young adulthood, included relatively few participants with ADHD (*n* = 19) (42). Thus, there is a need for studies examining developmental trajectories over a longer time-span and including several assessment waves and more participants with ADHD.

### Development of Co-occurring Symptoms in ASD

High levels of co-occurring symptoms are found across the lifespan in individuals with ASD (5, 13, 29, 43, 44). However, few studies have examined the developmental trajectories of co-occurring symptoms from childhood to adulthood in individuals with ASD (29). In a recent review of longitudinal studies from childhood to adolescence, the authors concluded that the literature suggests a slightly decline in co-occurring symptoms from childhood to adolescence in individuals with ASD, despite persistent higher rates compared with TD individuals (45). This conclusion is in line with previous findings by our research group, showing a decline in parent-reported, but not self-reported, co-occurring symptoms over a 2-year period (25, 43).

A few studies investigating the developmental trajectories of co-occurring symptoms form childhood to young adulthood in individuals with ASD have been conducted. Two studies have found declining trajectories of internalizing and externalizing behaviors from childhood (mean age 8 and 12 years, respectively) to young adulthood (mean age 24 and 23 years, respectively) in individuals with ASD (46, 47). In contrast, another study reported an increase in internalizing behaviors from childhood (age 9) to young adulthood (age 24) in individuals with ASD (48). McCauley et al. (49) identified different trajectories of internalizing behaviors (anxiety and depression symptoms, respectively) from childhood (mean age 9 years) to young adulthood (mean age 20 years) in individuals with ASD. The authors identified one group with stable low anxiety symptoms and one group with stable high anxiety symptoms. Similarly, the authors identified one group with stable low depressive symptoms and one group with fluctuating but stable high depression symptoms. Thus, two studies have suggested declining trajectories of internalizing and externalizing behaviors (46, 47), one study have suggested stable trajectories of internalizing behaviors (49), and one study have suggested increasing trajectories of internalizing behaviors (48) from childhood to young adulthood among individuals with ASD.

One limitation with the four aforementioned longitudinal studies were the inclusion of individuals with ASD both with and without intellectual disability. In fact, in all four cohorts the majority of participants have been reported to have an intellectual disability (IQ < 70) (46, 48, 50, 51). Thus, there is a need for studies focusing only on individuals with ASD without intellectual disability. This is because higher IQ is associated with different trajectories of co-occurring symptoms (49). For example, McCauley et al. (49) found that higher childhood verbal IQ was associated with greater odds of being in the high/stable trajectory of anxiety symptoms and the high-fluctuating trajectory of depressive symptoms. Further, using the same trajectory measure with individuals with and without intellectual disability cloud the picture, because measures intended for individuals without intellectual disability may be poor measures of co-occurring symptoms among individuals with intellectual disability, and vice versa (49, 52). Another limitation of the aforementioned studies is that none of them included a TD control group for comparison of trajectories. Nonetheless, Gray et al. (46) and Stringer et al. (47) compared co-occurring symptoms in adulthood with a normative sample or clinical norms. They found that, despite a decline in co-occurring symptoms from childhood to adulthood, individuals with ASD still displayed high-levels of co-occurring symptoms when compared with normative data. This is in line with studies finding a relatively high prevalence of co-occurring symptoms among adults with ASD (13, 17, 53, 54). In conclusion, the current literature suggests that individuals with ASD display declines in co-occurring symptoms from childhood to adulthood, but continue to display high levels of co-occurring symptoms in adulthood.

### Knowledge Gaps

To our knowledge, no study has previously compared the developmental trajectories of co-occurring symptoms from childhood to young adulthood in individuals with ADHD and individuals with ASD. Further, only one previous study (with few participants) have examined the trajectories of co-occurring symptoms in individuals with ADHD from childhood to young adulthood (42), and no previous study has examined the developmental trajectories of individuals with ASD exclusively without intellectual disability or included a TD comparison group. Thus, a study examining the developmental trajectories of individuals with ADHD and individuals with ASD without intellectual disability, and comparing their trajectories with TD individuals, is warranted. Further, longitudinal studies comparing the developmental trajectories of co-occurring symptoms in individuals with ADHD with individuals with ASD from childhood to young adulthood are needed in order to understand the unique and shared developmental trajectories of these two disorders. Similar developmental trajectories could suggest that communalities in genotype and/or endophenotypes (e.g., executive dysfunction; social cognitive difficulties) contribute to the developmental trajectories of co-occurring symptoms across both groups, whereas differential trajectories could suggest that the two disorders unique factors play an important role.

### Study Aims

We aimed to examine and compare the developmental trajectories of co-occurring symptoms among individuals with ADHD, individuals with ASD, and TD individuals from childhood to young adulthood. In line with the majority of previous studies, we hypothesized that individuals with ADHD and individuals with ASD would display declining trajectories of co-occurring symptoms over time (25, 40, 42, 45–47). Further, given that previous studies have suggested that ADHD and ASD are associated with high levels of co-occurring symptoms across the lifespan, we hypothesized that individuals with ADHD and individuals with ASD would continue to display higher levels of co-occurring psychopathology symptoms compared with TD individuals in young adulthood. Since we expected a decline in co-occurring symptoms in both individuals with ADHD and individuals with ASD, and these two groups share endophenotypes assumed to influence co-occurring symptoms, we hypothesized that they would display similar developmental trajectories.

## Methods

### Procedure and Participants

This study is part of the Lillehammer Neurodevelopmental Follow-up Study (LINEUP), a longitudinal study currently spanning baseline assessment (T1), 2-year follow-up (T2), and 10-year follow-up (T3). At T1, 85 children with ADHD, 38 children with ASD, and 50 TD children participated. Participants were recruited from child and adolescent psychiatric outpatient clinics at Innlandet Hospital, Norway, upon consecutive referrals. All individuals between 8 and 17 years of age referred for assessment of ADHD or ASD were invited to participate in the study. Those agreeing to participate were assessed for ADHD or ASD and included in the study if they meet diagnostic criteria based on DSM-IV. Thus, age at T1 equals the time of diagnosis in our sample. TD individuals were recruited through local schools and had to attend regular classes. At T1, all TD individuals were screened for mental disorders with the Kiddie-Schedule for Affective Disorders and Schizophrenia/Present and lifetime version (Kiddie-SADS), conducted separately with participants and their parents. Exclusion criteria for all participants were prematurity (<36 weeks), having a disease affecting the central nervous system, or having IQ < 70. An additional exclusion criterion for the ADHD group was no history of stimulant treatment. Additional criteria for the TD group were no history of psychiatric disorder, dyslexia or head injury with loss of consciousness. Demographic and clinical characteristics of all three groups are presented in Table 1. There were no significant differences in age or gender between the groups, but the groups differed on IQ, mothers' education, and ASD/ADHD symptomology.

**Table 1:** Demographic and clinical characteristics of the three participant groups.

	**ADHD**^**1**^ ***n*** **=** **85**		**ASD**^**2**^ ***n*** **=** **38**		**TD**^**3**^ ***n*** **=** **50**		**Group difference**	
**T1—baseline**	***M***	***SD***	***M***	***SD***	***M***	***SD***	***p***	**Bonferroni**
Age	11.61	2.08	12.03	2.34	11.56	1.99	0.532	
% male/female	54/46	84/16	64/36	0.006				
Full scale IQ	94.44	13.75	98.26	17.82	103.78	12.95	**0.002**	1 <3
Mothers' education in years	12.66	2.15	12.79	2.67	14.58	2.37	** <0.001**	1, 2 <3
Autism Spectrum Screening Questionnaire	9.68	10.09	21.47	9.27	1.62	1.85	** <0.001**	3 <1 <2
ADHD Rating Scale IV	26.62	10.51	21.37	10.28	2.64	2.99	** <0.001**	3 <2 <1
CGAS	57.46	8.94	51.61	10.18			**0.002**	
**T2−2-year follow-up**								
Sample retention (%)	81 (95.3%)	37 (97.4%)	50 (100.0%)					
Age	13.64	2.14	14.22	2.35	13.62	1.95	0.342	
% male/female	53/47	84/16	64/36	0.006				
Autism Spectrum Screening Questionnaire	7.68	7.26	20.56	9.21	1.04	2.78	** <0.001**	3 <1 <2
ADHD Rating Scale IV	18.18	10.91	14.44	8.70	2.22	2.70	** <0.001**	3 <1, 2
**T3−10-year follow-up**								
Sample retention (%)	61 (71.8%)	26 (68.4%)	40 (80%)					
Age	21.38	2.25	22.15	2.62	20.88	1.88	0.078	
% male/female	56/44	81/19	65/35	0.014				

*ADHD, attention-deficit/hyperactivity disorder; ASD, autism spectrum disorder; TD, typically developing. The bold values indicate a significant p-value after Bonferroni correction. Superscript values indcates the group number used in the Bonferroni column*.

#### Diagnostic Assessment

Diagnostic assessment at T1 was based on a semi-structured clinical interview (Kiddie-SADS) (55) conducted separately with participants and their parents, and supplemented with information from the Autism Spectrum Screening Questionnaire (ASSQ) (56) and the ADHD Rating Scale IV [ARS-IV; (69)]. All measures have demonstrated good reliability and validity (55–59). Diagnostic assessment was conducted by experienced clinical psychologists and clinical educational therapist trained to achieve high interrater reliability on diagnostic assessments. Diagnostic decisions were based on comprehensive evaluations of information from the Kiddie-SADS, ASSQ, ARS-IV, and teacher reports on academic and social functioning. The assessments and diagnostic decisions were supervised and independently reviewed by a clinical psychologist specialized in neurodevelopmental disorders (M. Ø.). Disagreements were discussed in order to arrive at a “best estimate” DSM-IV consensus diagnosis. Eight participants meet the criteria for both ADHD and ASD, and were included in the ASD group. In the ADHD group, three participants had comorbid depressive disorder, one had separation anxiety, one had specific phobia, one had social anxiety disorder, three had generalized anxiety disorder, five had Tourette's syndrome, two had conduct disorder, and nine had oppositional defiant disorder. In the ASD group, two had comorbid depressive disorder, one had panic disorder, one had separation anxiety, three had specific phobias, two had social anxiety disorder, one had generalized anxiety disorder, one had obsessive-compulsive disorder, four had Tourette's syndrome, one had conduct disorder, and one had oppositional defiant disorder.

#### Treatment as Usual

Participants with ADHD and ASD received treatment as usual (TAU) at the child and adolescent psychiatric outpatient clinic after diagnosis. TAU could include psychoeducation for parents, medical treatment, and in some instances unstructured social skills training (for the ASD group). Participants were generally referred for treatment whiteout explicit focus on co-occurring symptoms, and thus, clinical focus may have centered on core symptoms and school performances and less on co-occurring symptoms (43). At T2, 28 participants (35%) in the ADHD group and 15 (41%) in the ASD group had a statement of special needs education, meaning that they received some kind of extra support at school. In the ADHD group, 44 participants (54%) received stimulant medication at T2, and seven participants (12%) received stimulant medication at T3. In the ASD group, four participants (11%) received stimulant medication at T2, and two participants (8%) received stimulant medication at T3.

#### Sample Retention

Two- and 10-years after baseline assessment, all participants were invited for follow-up assessment. Out of the 173 original participants, 168 participated at T2, giving an overall retention rate of 97.1% (see Table 1 for retention rates in each group). At T3, 127 of the original participants were re-assessed, giving an overall retention rate of 73.4%. Of the 46 original participants not re-assessed at T3, we were unable to track down nine of them (four from the ADHD group; three from the ASD group; two from the TD group) and 37 declined further participation (20 from the ADHD group; 9 from the ASD group; 8 from the TD group). We examined differences between those who participated at T3 and those dropping-out in baseline (T1) characteristics; age, gender, full scale intelligence quotient, mothers' educational level, symptom severity (ASSQ; ARS-IV), and psychopathology symptoms (Child Behavior Checklist, total problems subscale). We used one-way analysis of variance (ANOVA) and chi-square test of independence with Bonferroni corrected alpha-level (0.05/7 = 0.007). There was no statistically significant difference between the two groups in any of the seven baseline characteristics (all *p* ≥ 0.013).

### Measures

#### Psychopathology Symptoms

We used the Achenbach System of Empirically Based Assessment (ASEBA) (60, 61) as a primary measure of psychopathology symptoms across all three waves. We chose the ASEBA because it is standardized across all ages, widely used in clinical settings, and includes a validated Norwegian translation (62, 63).

At T1 and T2, we used the ASEBA scale, Child Behavior Checklist (CBCL) (60), a parent reported scale comprising 113 items assessing psychopathology symptoms along eight syndrome scales (anxious/depressed; withdrawn/depressed; somatic complaints; social problems; thought problems; attention problems; rule-breaking behavior; aggressive behavior). In addition, the CBCL includes two broader band scales of internalizing behaviors (comprising anxious/depressed, withdrawn/depressed, and somatic complaints) and externalizing behaviors (comprising rule-breaking and aggressive behavior), as well as a total problems scale (comprising all eight syndrome scales). Raw scores are converted to *T*-scores (M = 50, SD = 10) based on American norms. Norwegian norms does not exist, but Norwegian children generally seems to obtain lower *T*-scores compared with American children (64). The psychometric properties of the CBCL are good regarding both reliability (α ≥ 0.80), sensitivity (40–83%), and specificity (70–94%), and confirmatory factor analyses have confirmed the factor structure across different countries (62–65).

At T3, we used the ASEBA scale, Adult Self-Report (ASR) (61), a self-report scale comprising 126 items assessing psychopathology symptoms along eight syndrome scales. These syndrome scales are the same as for the CBCL with one exception, the social problems subscale is replaced by an intrusiveness subscale. As for the CBCL, the syndrome scales are included in two broader band scales of internalizing behaviors (comprising anxious/depressed, withdrawn/depressed, and somatic complaints) and externalizing behaviors (comprising rule-breaking, aggressive, and intrusive behavior), as well as a total problems scale (comprising all syndrome scales). Raw scores are converted to age-adjusted T-scores, making it easy to compare within individual scores over time. The ASR have demonstrated good reliability (α ≥ 0.81) and validity (66–68).

#### ADHD Symptomatology

At T1 and T2, the ARS-IV (69) was used as measure of ADHD symptomatology. The ARS-IV is a parent reported scale comprising 25 items assessing ADHD symptoms across the two domains; hyperactivity/impulsivity and inattention. The ARS-IV has demonstrated good psychometric properties with good test-retest reliability of ≥0.77 and internal consistency (α ≥ 0.80) (58, 70).

#### ASD Symptomatology

At T1 and T2, the ASSQ (56) was used as measure of ASD symptomatology. The ASSQ is a parent reported scale comprising 27 items assessing ASD symptoms across social interaction problems, communication, and restricted and repetitive behaviors. The ASSQ has demonstrated good psychometric properties with good test-retest reliability of 0.96 and convergent validity with other measures of ASD symptomatology (56).

### Statistical Analyses

We used SPSS version 26 for all statistical analyses. We used an alpha-level of 0.05 for statistical significance and used Bonferroni corrected alpha-levels for multiple comparisons. To examine differences in demographic and clinical variables between groups, we used analysis of variance (ANOVA) and chi-square test of independence. Differences between the ADHD, ASD, and TD groups were considered significant if *p*-values were below 0.004 (0.05/13 = 0.004).

To examine the developmental trajectories of co-occurring symptoms and relate changes over time to group affiliation, we used Linear Mixed Models (LMM) for longitudinal analyses. The technique includes participants with partial data and is thus more robust in longitudinal studies where missing data is common (71, 72). We estimated the model using restricted maximum likelihood, with piecewise linear splines, with a knot at T2. We fitted separate random intercepts and slopes for the first (T1–T2) and second (T2–T3) time period. Fixed effects of group x time interactions were the parameters of main interest, indicating differences in trajectories between groups. To assess all three group comparisons, we fitted models with all three groups (TD vs. ADHD/ASD) and with only ADHD vs. ASD. We used the Akaike information criteria to assess model fit. We used Pearson's correlation-coefficient, *r*, to examine associations between changes in core symptoms (ADHD and ASD) and changes in co-occurring symptoms from T1 to T2.

To examine group differences in outcome (i.e., psychopathology symptoms at T3), we used multivariate analysis of variance (MANOVA) with the three ASEBA scales internalizing behaviors, externalizing behaviors, and total problems as dependent variables. A significant group effect (Wilks Lambda) was followed-up with ANOVAs for each dependent variable and Bonferroni *post-hoc* tests.

## Results

### Trajectories of Co-occurring Psychopathology Symptoms

**Figure 1:**
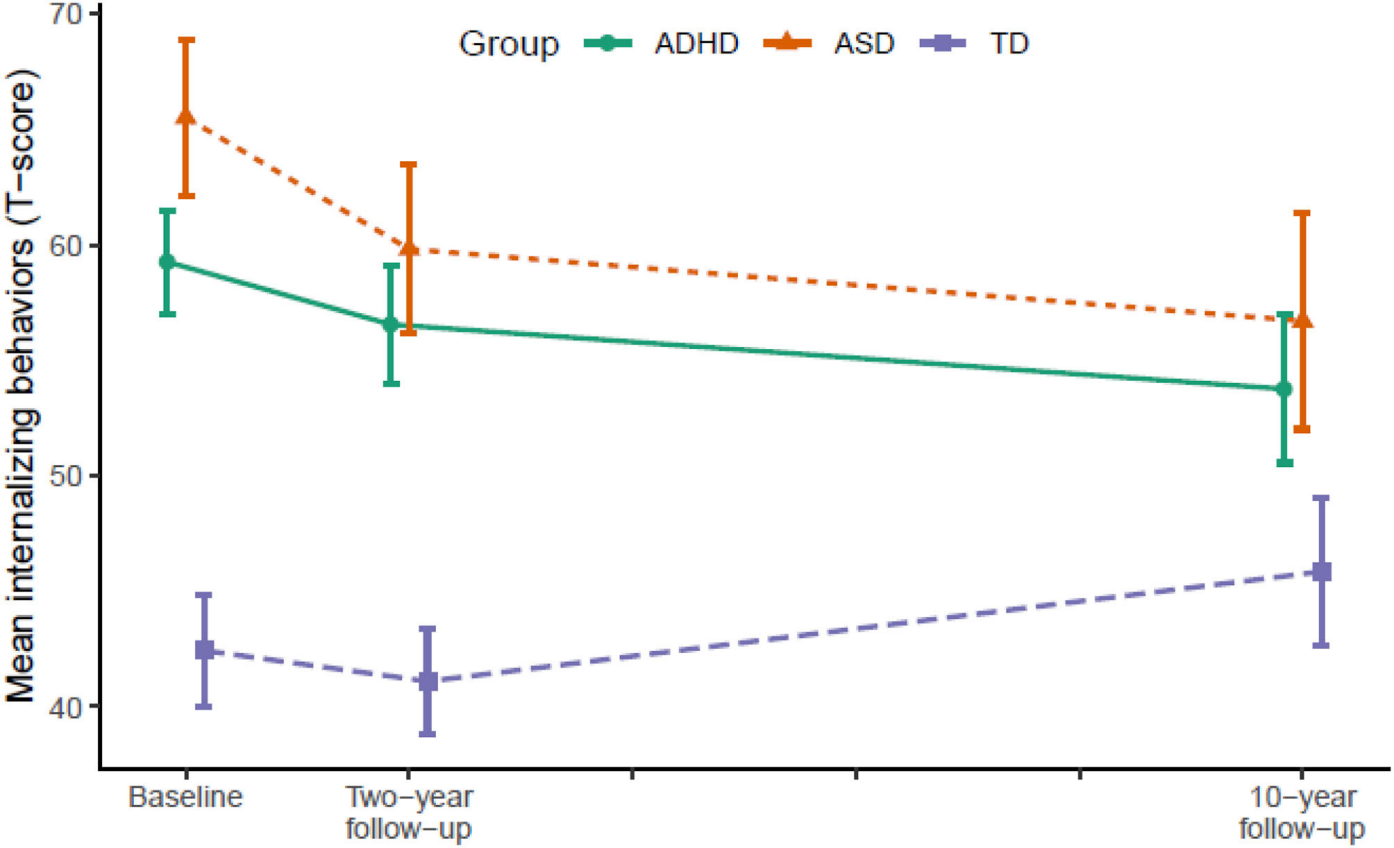
Developmental trajectories of internalizing behavior from baseline (T1) to 10-year follow-up (T3). Vertical error bars indicate 95% CI; Groups are dodged slightly along x-axis to prevent over plotting; Internalizing behaviors = Achenbach System of Empirically Based Assessment; ADHD, attention-deficit/hyperactivity disorder; ASD, autism spectrum disorder; TD, typically developing.

**Figure 2:**
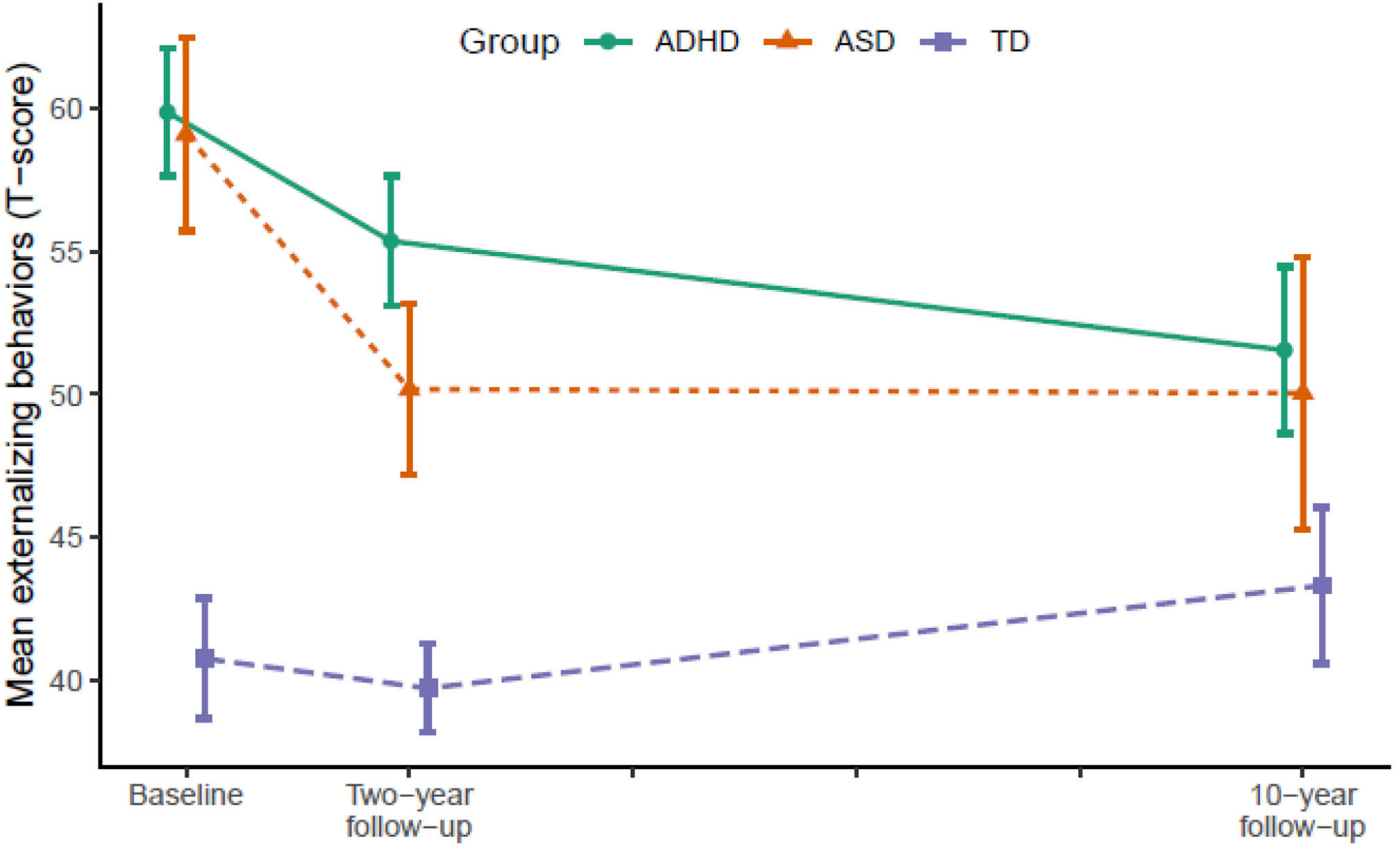
Developmental trajectories of externalizing behavior from baseline (T1) to 10-year follow-up (T3). Vertical error bars indicate 95% CI; Groups are dodged slightly along x-axis to prevent over plotting; Externalizing behaviors = Achenbach System of Empirically Based Assessment; ADHD, attention-deficit/hyperactivity disorder; ASD, autism spectrum disorder; TD, typically developing.

**Figure 3:**
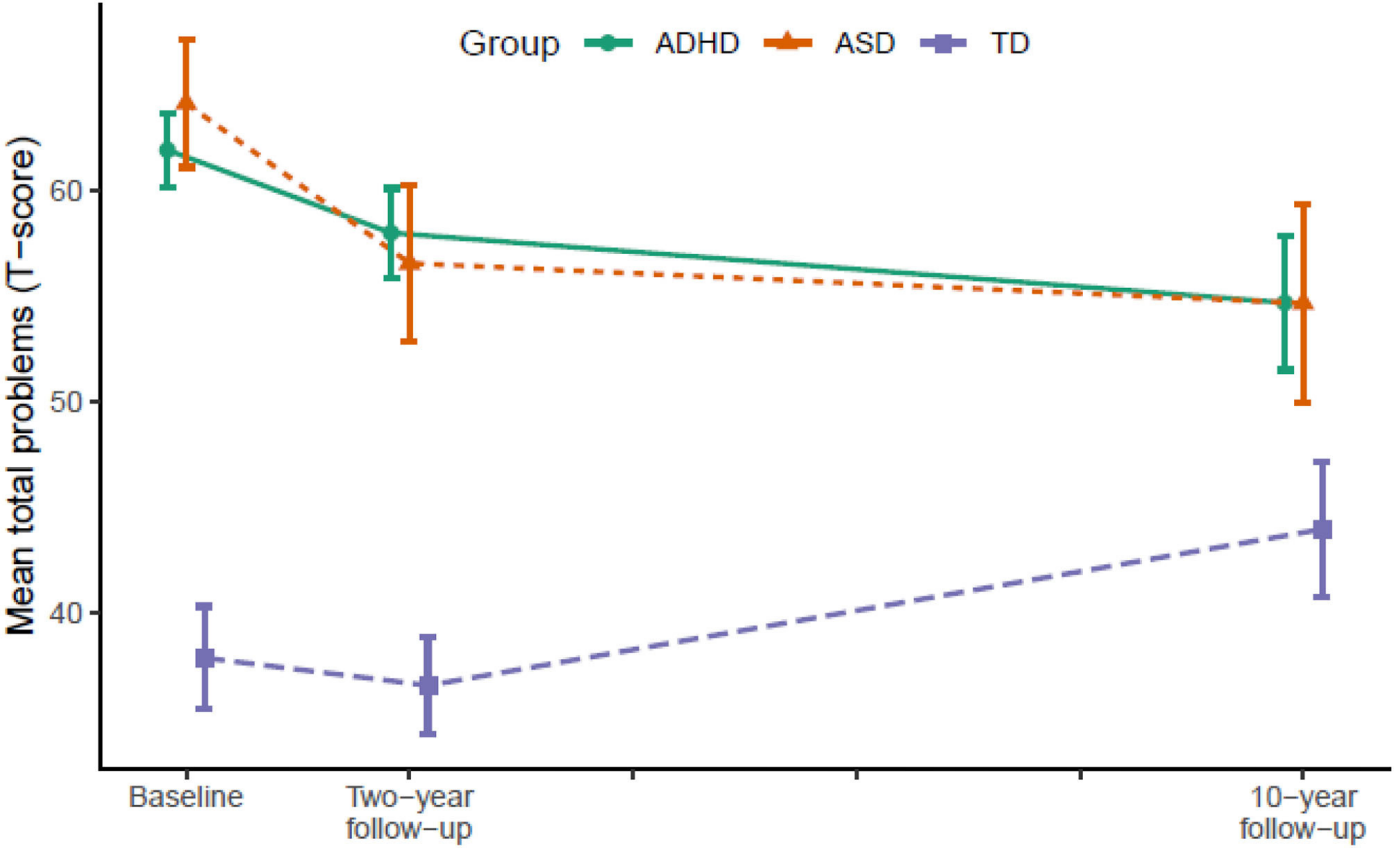
Developmental trajectories of total problems from baseline (T1) to 10-year follow-up (T3). Vertical error bars indicate 95% CI; Groups are dodged slightly along x-axis to prevent over plotting; Total problems = Achenbach System of Empirically Based Assessment; ADHD, attention-deficit/hyperactivity disorder; ASD, autism spectrum disorder; TD, typically developing.

Table 2 displays the results from LMM for each symptom trajectory and Figures 1–3 display the trajectories of the three groups across the dependent variables. At baseline (T1), the ADHD and the ASD group displayed significantly higher levels of psychopathology symptoms than the TD group (Table 2). The ASD group displayed significantly higher levels of internalizing behaviors compared with the ADHD group, whereas there was no significant difference between the ADHD and the ASD group in externalizing behaviors and total problems. From T1 to T2, the ADHD group displayed a significant decline (*p* = 0.025) in externalizing behaviors relative to the TD group, whereas the ASD group displayed significant declines in both internalizing behaviors (*p* = 0.036), externalizing behaviors (*p* < 0.001), and total problems (*p* = 0.001) relative to the TD group. The declines in externalizing behaviors (*p* = 0.020, −4.5 *T*-scores) and total problems (*p* = 0.047, −3.6 *T*-scores) were significantly larger in the ASD group compared with the ADHD group.

**Table 2:** Fixed effects of time and group in a Linear Mixed model, with follow-up over 10 years in ASD, ADHD, and TD groups.

**ASEBA scale**	**Internalizing**			**Externalizing**			**Total problems**		
	**Estimate**	**SE**	**95% CI**	**Estimate**	**SE**	**95% CI**	**Estimate**	**SE**	**95% CI**
**Main effect of group**									
TD	0 (ref)			0 (ref)			0 (ref)		
ADHD	16.92^***^	1.80	13.37, 20.48	19.02^***^	1.75	15.57, 22.46	24.02^***^	1.56	20.96, 27.07
ASD	23.18^***^	2.17	18.90, 27.46	18.25^***^	2.10	14.10, 22.40	26.23^***^	1.87	22.55, 29.91
ADHD (ref) vs. ASD	6.26^**^	2.06	2.17, 10.34	−0.77	2.05	−4.83, 3.29	2.21	1.69	−1.12, 5.55
**Main effect of time**									
T1–T2	−1.25	1.33	−3.88, 1.38	−1.09	1.23	−3.52, 1.34	−1.32	1.23	−3.74, 1.10
T2–T3	4.85^*^	2.18	0.54, 9.16	3.62	1.98	−0.29, 7.53	7.47^***^	2.15	3.21, 11.72
**Interaction group** **×** **time (T1–T2)**									
TD	0 (ref)			0 (ref)			0 (ref)		
ADHD	−1.49	1.69	−4.82, 1.85	−3.52^*^	1.56	−6.59, −0.45	−2.72	1.56	−5.80, 0.35
ASD	−4.38^*^	2.07	−8.47, −0.29	−7.97^***^	1.90	−11.73, −4.21	−6.32^***^	1.91	−10.09, −2.54
ADHD (ref) vs. ASD	−2.89	1.98	−6.82, 1.04	−4.45^*^	1.89	−8.19, −0.72	−3.58^*^	1.79	−7.12, −0.05
**Interaction group** **×** **time (T2–T3)**									
TD	0 (ref)			0 (ref)			0 (ref)		
ADHD	−7.65^**^	2.81	−13.21, −2.09	−7.45^**^	2.55	−12.49, −2.41	−10.77^***^	2.15	−16.26, −5.28
ASD	−8.19^*^	3.54	−15.19, −1.20	−3.91	3.21	−10.23, 2.42	−9.55^**^	3.49	−16.44, −2.65
ADHD (ref.) vs. ASD	−0.38	3.71	−7.74, 6.98	3.62	3.34	−3.00, 10.24	1.30	3.41	−5.45, 8.05

*Internalizing, externalizing, and total problems = Achenbach System of Empirically Based Assessment; ADHD, attention-deficit/hyperactivity disorder; ASD, autism spectrum disorder; TD, typically developing; T1, baseline; T2, 2-year follow-up; T3, 10-year follow-up*.

* *p < 0.05*,

** *p < 0.01*,

*** *p < 0.001*.

From T2 to T3, the ADHD group displayed significant declines in internalizing behaviors (*p* = 0.007), externalizing behaviors (*p* = 0.004), and total problems (*p* < 0.001) relative to the TD group. The ASD group continued to display declines in internalizing behaviors (*p* = 0.022) and total problems (*p* = 0.007) relative to the TD group, but not externalizing behaviors. In contrast, internalizing behaviors (*p* = 0.028) and total problems (*p* = 0.001) increased significantly in the TD group.

In total from T1 to T3, the ADHD group displayed a small to medium (*d* = −0.49) decline in internalizing behaviors and medium to large declines in externalizing behaviors (*d* = −0.79) and total problems (*d* = −0.71). The ASD group displayed large declines in both internalizing behaviors (*d* = −0.79), externalizing behaviors (*d* = −0.80), and total problems (*d* = −0.89). From T1 to T2, the decline in externalizing behaviors in the ADHD group correlated significantly with a decline in ADHD symptoms (*r* = 0.45, *p* < 0.001) and the declines in internalizing (*r* = 0.35, *p* = 0.040) and externalizing behaviors (*r* = 0.51, *p* = 0.002) in the ASD group correlated significantly with a decline in ASD symptoms. The decline in total problems in the ASD group correlated non-significantly with a decline in ASD symptoms (*r* = 0.32, *p* = 0.062).

### Group Differences at T3

A MANOVA with ASEBA scales at T3 as dependent variables showed a significant effect of group [*F*_(6, 236)_ = 4.584, *p* < 0.001, $\eta_{\text{p}}^{2}$ = 0.104]. Separate ANOVAs for each dependent variable showed a significant group effect in all three domains (see Table 3). For internalizing behaviors, *post-hoc* tests showed that the ADHD (*p* = 0.004, *d* = 0.69) and the ASD (*p* = 0.002, *d* = 0.98) group displayed significantly more internalizing behaviors compared with the TD group. For externalizing difficulties, *post-hoc* tests showed that the ADHD group displayed significantly more externalizing behaviors compared with the TD group (*p* = 0.001, *d* = 0.80), whereas the ASD group displayed non-significantly more externalizing behaviors compared with the TD group (*p* = 0.051, *d* = 0.64). For total problems, *post-hoc* tests showed that the ADHD (*p* < 0.001, *d* = 0.94) and the ASD (*p* = 0.002, *d* = 0.97) group displayed significantly more total problems compared with the TD group. There were no significant differences between the ADHD and the ASD group.

**Table 3:** Group differences across ADHD, ASD, and TD in co-occurring symptoms at 10-year follow up (T3).

**ASR**	**ADHD**^**1**^ **(*****n*** **=** **59)**		**ASD**^**2**^ **(*****n*** **=** **24)**		**TD**^**3**^ **(*****n*** **=** **40)**				
	***M***	***SD***	***M***	***SD***	***M***	***SD***	**Group comparison**		**Bonferroni**
							***F***	***p***	
Internalizing^a^	53.76	12.61	56.71	11.72	45.85	10.38	8.038	0.001	1, 2 > 3
Externalizing^a^	51.54	11.44	50.04	11.89	43.33	8.81	7.241	0.001	1 > 3
Total problems^a^	54.69	12.45	54.67	11.74	43.98	10.29	11.417	<0.001	1, 2 > 3

a *Adult Self-Report, Achenbach System of Empirically Based Assessment; ADHD, attention-deficit/hyperactivity disorder; ASD, autism spectrum disorder; TD, typically developing. Superscript values indcates the group number used in the Bonferroni column*.

## Discussion

Our findings provide the field with at least three new pieces of knowledge. First, we provide evidence showing declining trajectories of co-occurring symptoms across three assessment waves from childhood to young adulthood (mean age 11–21) in individuals with ADHD. Second, we provide evidence suggesting similar but partially differential trajectories of co-occurring symptoms among individuals with ADHD and individuals with ASD. Third, we found that individuals with ADHD and individuals with ASD continue to display more co-occurring symptoms in young adulthood compared with TD individuals, despite differential developmental trajectories in the three groups (declining vs. increasing). Overall, our findings highlight the persistence of co-occurring symptoms among individuals with ADHD and ASD, respectively, while at the same time providing some optimism regarding the symptom trajectories.

The decline in co-occurring symptoms among individuals with ADHD is in accordance with our hypothesis, previous studies (42), and is consistent with the declining trajectory of ADHD symptomatology observed from childhood to adulthood (73–76). This may indicate that ADHD core symptomatology and co-occurring symptoms follows similar trajectories from childhood to young adulthood. Previous studies have suggested interdependence between ADHD core symptomatology and co-occurring symptoms [e.g., (77, 78)]. Our findings support this interdependence through demonstrating significant associations between changes in core symptomatology and co-occurring symptoms. Thus, it seems that declines in either ADHD symptomatology or co-occurring symptoms associates with declines in the other symptom domain. However, our research group has previously found that declines in ADHD symptoms over a 2-year period were differentially associated with a decline or increase in depressive symptoms in girls vs. boys and based on self-report vs. parent-report (78). Thus, further research examining the relationship between changes in co-occurring symptoms and ADHD symptomatology is needed to understand the interconnected development of these symptom domains. Further, studies examining predictors of change are warranted as this provide knowledge about what factors may be important to target in interventions.

Similar to the ADHD group, and in accordance with our hypothesis, we found declines in co-occurring symptoms across all domains for the ASD group. This is consistent with two of the previous studies of individuals with ASD (46, 47). Our findings suggest that also individuals with ASD without intellectual disability display declining trajectories of co-occurring symptoms from childhood to young adulthood. There were few differences in the trajectories of co-occurring symptoms from childhood to young adulthood between the ADHD and the ASD group. The ASD group displayed larger declines in externalizing behaviors and total problems from T1 to T2 compared with the ADHD group. Children with ASD without intellectual disability often receives the diagnosis late (around 11–12 years), typically after several years of diagnostic assessment (79). We therefore speculate on whether a late diagnosis of ASD and lack of ASD specific treatment and individual tailoring at school could contribute to this finding. When receiving the diagnosis, co-occurring symptoms among children with ASD are at its most extreme and from there only declines can be observed (i.e., regression toward the mean) (80). In addition, children with ASD may benefit from more individual tailoring at home and school after diagnosis. A diagnosis is often necessary to gain access to social skills training and support staff at school, factors contributing to declines in co-occurring symptoms among children with ASD (81–83). Consequently, considerable declines in co-occurring symptoms are observed after diagnosis.

In contrast to ASD, we speculate on whether the co-occurring symptoms among children with ADHD may be closer linked to the core symptoms (84) and that children with ADHD may have poorer access and benefit less from individual tailoring at school and psychological treatments such as social skills training (85, 86). Clinical guidelines for treatment of ADHD differs from guidelines for ASD with a greater emphasis on medical treatment and less emphasis on individual tailoring and psychological treatments, despite the fact that many children with ADHD can benefit from the same interventions as children with ASD (87, 88). In our sample, 54% of participants with ADHD received medical treatment at T2, and in accordance with clinical guidelines, all participants shall have received an offer of medical treatment. Thus, almost half of our participants either declined the offer or discontinued medical treatment during the first 2-year period following diagnosis. Since we did not focus on treatment in this study, the reasons for this are unknown, but it is interesting to note that the ADHD group, of which 54% received medical treatment between T1 and T2, had less declines in co-occurring symptoms than the ASD group, which probably received other forms of intervention. However, based on our results we can only speculate to the reasons for this difference, and hence, more research is needed to understand which factors drive the larger declines in co-occurring symptoms from childhood to adolescence in the ASD group compared with the ADHD group. It would be interesting in the future to include variables related to school support and interventions as well as parental adaptations at home after receiving the diagnosis to examine whether these factors differentially influence children with ADHD and children with ASD.

A finding that was particularly notable when comparing the developmental trajectories of the ADHD and ASD group with that of the TD group, was that the ADHD and the ASD group displayed declines in co-occurring symptoms from T2 to T3 (mean age 13–21), a period when the TD group displayed increases in internalizing behaviors and total problems. This suggests that while researchers and clinicians have been worrying that individuals with ADHD and ASD may be particularly vulnerable during the transitional periods of adolescence and young adulthood (89, 90), the opposite may be true. Individuals with ADHD or ASD may experience less distress during the periods of adolescence and young adulthood compared with childhood. Perhaps because the transitional periods of adolescence and young adulthood offers opportunities for a new start, greater autonomy in social and academic situations, opportunities to pursue own interests, and a more inclusive environment for instance at high school and university [e.g., (91)]. However, it could also be that these transitional periods provoke other forms of co-occurring symptoms, not measured in the current study, such as substance-abuse, criminal behavior, and personality disorders (42, 92).

Another explanation may be that individuals with ADHD and individuals with ASD have an illusory self-perception (93, 94). Some studies have found that individuals with ADHD or ASD have a tendency to underreport social, emotional, and cognitive difficulties compared with parental reports (93–96). This could suggest that individuals with ADHD and individuals with ASD lack self-awareness about own difficulties, and thus, the decline observed from T2, with the use of parent-report, to T3, with the use of self-report, could be partially attributed to an illusory self-perception causing young adults with ADHD or ASD to underreport their co-occurring symptoms. Indeed, previous studies including both parent- and self-report have observed such discrepancy in young adulthood (37, 97).

Despite considerable declines in co-occurring symptoms from childhood to young adulthood in the ADHD and the ASD group, both groups continued to display elevated levels of co-occurring symptoms relative to the TD group in young adulthood. The ADHD group displayed more co-occurring symptoms across all domains, whereas the ASD group displayed more internalizing behaviors and total problems compared with the TD group. It should be noted that both the ADHD and the ASD group had scores around or only slightly above the normed mean of 50 (*T*-score) at T3, suggesting a low load of co-occurring symptoms relative to the US general population. However, previous studies have suggested that Norway is a low scoring country where the population mean is well below 50 (64). Therefore, the comparison with our TD group provides a more context sensitive comparison of co-occurring symptoms. Thus, the results provide evidence for the persistence of co-occurring symptoms among individuals with ADHD and individuals with ASD, and underscores the importance of early efforts to treat and prevent co-occurring symptoms. Addressing co-occurring symptoms in childhood may improve developmental trajectories, which in turn could benefit adult functioning and quality of life [e.g., (20, 22, 24)].

### Strengths and Limitations

The LINEUP study is unique by the inclusion of individuals with different neurodevelopmental disorders (i.e., ASD; ADHD) ascertained after careful diagnostic assessment including clinical interview, self-, parent-, and teacher-report of social, emotional, and academic functioning, and neuropsychological tests (16). To our knowledge, no other longitudinal study spanning childhood to young adulthood (i.e., 10 years) have included several types of neurodevelopmental disorders. Thus, our study is to date the only study allowing comparison of the developmental trajectories of co-occurring symptoms across different neurodevelopmental disorders. Other strengths include a relatively high sample retention and the use of the same measurement system of co-occurring symptoms across all waves (i.e., the ASEBA).

Despite the study's notable strengths, some limitations should be mentioned. One limitation is the use of parent-report at T1 and T2 and self-report at T3. Previous studies of individuals with ADHD or ASD have suggested that young adults may be underreporting externalizing behaviors compared with parental reports (37, 97), which can contribute to the decline observed from T2 to T3. However, whereas externalizing behaviors may be underreported by young adults themselves, internalizing behaviors may be underreported by parents (98, 99), and obtaining valid parental reports can be difficult when young adults are living independently of their parents (97). Thus, an underreporting of externalizing behaviors at T3 is possible, but we think it strengthens the validity of our results that, despite this possible underreporting, we found moderate to large differences in externalizing behaviors when comparing the ADHD and the ASD group to the TD group at T3 (*d* = 0.64–80), and externalizing behaviors showed a similar developmental trajectory as internalizing behaviors and total problems.

A second limitation is that we did not obtain detailed information about possible treatment of co-occurring symptoms and other interventions the participants may have received between assessments. We assume that potential interventions in childhood are largely focused on school performance, activities of daily life, and social skills, rather than co-occurring symptoms *per se* (43). Still, we cannot completely rule out that some participants have received treatment targeting co-occurring symptoms. Further, interventions targeting social skills, school performance, and adaptive functioning may also have beneficial effects on co-occurring symptoms. However, participants in this study only received TAU and it would be ethically questionable to decline participants TAU.

A couple of limitations regarding our sample should also be noted. One limitation is that the sample comprised clinically referred individuals only, and thus, results may not be generalizable to the whole population of individuals with ADHD or ASD. A second limitation is the small proportion of females in the ASD group that, although consistent with the literature (100), prevented us from disentangling sex differences in the development of co-occurring symptoms. Studies have suggested that there may be sex differences in co-occurring symptoms in both individuals with ADHD and ASD [e.g., females displaying more internalizing behaviors than males; see (101, 102) for reviews]. Thus, sex differences in developmental trajectories of co-occurring symptoms should be investigated in future studies.

### Clinical Implications

Increased understanding of the developmental trajectories of co-occurring symptoms among individuals with ADHD and individuals with ASD has clinical implications for assessment and intervention. Our findings suggest that clinicians should assess and carefully monitor co-occurring symptoms in individuals with ADHD or ASD to detect clinically significant changes and to target interventions. Standard treatment for individuals with ADHD or ASD should include interventions to prevent and treat co-occurring symptoms and not exclusively focus on core symptoms, school performance, and adaptive functioning. Although these are important skills, and also related to co-occurring symptoms. Given the persistence of co-occurring symptoms among individuals with ADHD or ASD, interventions should be initiated as early as possible, preferably once a child receives the diagnosis (e.g., preventive interventions such as training in emotional control). Since individuals with ADHD seems to have a somewhat slower decline in co-occurring symptoms from childhood to adolescence compared with individuals with ASD, interventions targeting co-occurring symptoms in children and adolescents with ADHD may be especially important to expedite declining trajectories. It is also important that clinicians, parents, and teachers are aware of the high levels of co-occurring symptoms among these individuals, and the persistence of these symptoms over time. Parents may take comfort in knowing that co-occurring symptoms wane over time, but declining trajectories should not be used by clinicians as an excuse not to provide interventions.

## Data Availability Statement

The datasets presented in this article are not readily available because the data serving as the basis for the article submitted is stored in a secured repository at Innlandet Hospital Trust (Norway). Due to ethical restrictions on access to the data pursuant to the consent statements participants signed upon collecting the data, the authors are not permitted to upload a data set to sites outside of the repository. Access to the data, however, is available upon request to all serious researchers by contacting the following persons at Innlandet Hospital Trust: Erik Winther Skogli. Requests to access the datasets should be directed to erik.winther.skogli@sykehuset-innlandet.no.

## Ethics Statement

The studies involving human participants were reviewed and approved by Regional Committee for Medical Research Ethics in Eastern Norway. Written informed consent to participate in this study was provided by the participants' legal guardian/next of kin.

## Author Contributions

SO contributed to the conceptualization, data curation, formal analysis, and wrote the original draft. MØ contributed to the methodology, investigation, funding acquisition, supervision, and writing—reviewing and editing. IF contributed to investigation and data curation. PA contributed to methodology, investigation, supervision, and writing—reviewing and editing. ES contributed to conceptualization, methodology, investigation, formal analysis, funding acquisition, supervision, project administration, and writing—reviewing and editing. All authors approved the final manuscript for submission.

## Funding

This work was supported by grants from Innlandet Hospital Trust (Grant Numbers: 150610, 150624, and 150648) and from NevSom, Department of Rare Disorders and Disabilities, Oslo University Hospital (Grant Number: 150616).

## Conflict of Interest

The authors declare that the research was conducted in the absence of any commercial or financial relationships that could be construed as a potential conflict of interest.

## Publisher's Note

All claims expressed in this article are solely those of the authors and do not necessarily represent those of their affiliated organizations, or those of the publisher, the editors and the reviewers. Any product that may be evaluated in this article, or claim that may be made by its manufacturer, is not guaranteed or endorsed by the publisher.
